# Exposure to indoor tanning in France: a population based study

**DOI:** 10.1186/1471-5945-13-6

**Published:** 2013-04-23

**Authors:** Tarik Benmarhnia, Christophe Léon, François Beck

**Affiliations:** 1 National Institute for Prevention and Health Education (INPES), 42, Bld de la Libération, St Denis Cedex 93203, France

**Keywords:** Artificial UV radiation, Survey, Random sampling, Exposure, Opinions, Knowledge, Beliefs

## Abstract

**Background:**

Tanning lamp sessions have increased in Europe in recent years. Recent epidemiological studies have confirmed a proven link between melanoma and artificial UV exposure. However, in France, little information is available to determine the exposure of the population. This article presents the results from the ‘Baromètre cancer 2010’ concerning the proportion of users exposed to artificial UV radiation in France, their characteristics and level of information on the risks associated.

**Methods:**

A two stage random sampling telephone survey assisted by CATI system (household, individual) was performed from 3 April 2010 to 7 August 2010 on a sample of 3,359 people aged 15 to 75 years old.

**Results:**

In 2010, 13.4% of the French population reported to have tanning lamp sessions at least once in their lifetime and 3.5% of the total population reported the use of artificial UV radiation over the last twelve months. Exposure over the last twelve months is most commonly seen among females (5.0%) and young population between 20–25 years old (9.6%). In addition, 3.5% of those under 18 years report having attended UV booths at least once during their lifetime even though they are forbidden to minors. Moreover, more than one the third of users reported more than 10 exposures within a year. The places of exposure cited most often were beauty salons (50%) and tanning centers (46%). Only 49.2% of those surveyed felt that they were well informed on the risks of cancer associated with UV booths. Furthermore, the population was found to have misconceptions about artificial UV radiation. One quarter of the population, believe that artificial UV radiation use before vacation protects the skin from sunburn.

**Conclusions:**

This first study on artificial UV radiation exposure in France has better quantified and characterized the users. It has also defined the state of knowledge and the perception of risk by the general French population. This work will contribute to determine actions of prevention to reduce cancer risk related to artificial UV radiation.

## Background

The western population’s craze for sun since the 1980s has led to the rapid expansion of tanning sessions. The market for artificial UV tanning is not as large in France as in other European countries (Germany, Italy and Scandinavian countries).

This sector seems nonetheless poised to grow significantly in the future. A 2010 census estimated nearly 18,000 listed UV booths nationwide [1].

In 2004, in a telephone survey conducted by the National Institute for Prevention and Health Education (INPES) with a sample of 1002 people aged 15 and over, 55% of French people said they like to be tanned; 19% reported frequent sun exposure, 17% used cosmetics "tanning accelerators" (Monoi, oil, etc..) and 3% made tanning lamp sessions [2].

Skin cancers (basal cell, carcinoma, squamous cell carcinoma and melanoma) are the most common cancers, with nearly 80,000 new cases a year in France. They are also among the types of cancer that have increased the most over the last 50 years. The most severe form called cutaneous melanoma has seen its number of new cases triple between 1980 and 2005 [3]. According to the International Agency for Research on Cancer (IARC), nearly 70% of cutaneous melanoma cases are caused by sun exposure [4]. Recent epidemiological studies have confirmed a proven link between melanoma and artificial UV exposure. An IARC meta-analysis published in 2006 found the risk of melanoma is increased by 75% when the first exposure is before the age of 35 years (RR = 1.75 with CI 95%: 1.35-2.26) [5]. Moreover, two recent systematic reviews and meta-analysis found that sunbed use is associated with a significant increase in risk of melanoma, basal and squamous cell skin cancer [6,7]. They also only found that the risk is higher with use in early life.

Advances in scientific knowledge on the carcinogenic effects of UV rays (UVA and UVB) led the IARC, in July 2009, to include artificial UV radiation in the group of confirmed carcinogens for humans (group 1), just like radiation from the sun [8].

These last years, some studies have reported the frequency of tanning bed use in different contexts [9,10]. They reported the prevalence of tanning bed use, individual characteristics associated with this practice. For example, Börner et al. [9], found positive relationships of appearance and health related beliefs with tanning bed use and some misconceptions in users about the positive effect of artificial UV radiation A systematic literature review reported that users are characterized by a lack of knowledge about health risks of UV, and prompted by the frequent use of sunbeds by friends or family members and the experience of positive emotions and relaxation by indoor tanning [11]. These characteristics are context dependant however [12], and it is important to study specific user profiles in the French context, given the fact that today, limited data are available to accurately describe the frequency of use of UV booths in France and user profiles. Level of knowledge about the risks associated with artificial UV radiation as well as the reasons that bring a certain clientele to use UV booths are poorly documented. For the first time as part of this study, the French population was surveyed on its use of tanning sessions. The first goal was to determine the proportion of French users of artificial UV radiation as well as their characteristics and level of knowledge of the risks associated with artificial UV use. Some local studies have explored beliefs and practices about tanning bed use in France, but never in a population-based study [13,14]. This study provides a first look at the national current situation among 15–75 year olds, which will eventually help us to evaluate changes in exposure of the population as a result of future regulatory, information or risk awareness actions.

## Methods

The ‘Baromètre Cancer 2010’ is a two-stage random sampling survey (household then individual) performed using a *Computer-Assisted Telephone Interviewing* (CATI) system. The survey, assigned to GFK-ISL Institute, was carried out from 3 April to 7 August 2010 [15].

For a survey frame as exhaustive as possible, ‘Baromètre Cancer 2010’ has integrated, in addition to households with a fixed line (even those ex-directory), households contactable by mobile phone only and those with unbundled ADSL access. Phone numbers were generated randomly in order to be able to interview households corresponding to unlisted phone numbers. The study protocol included a formal request to participate, explaining the objectives of the study that was delivered by mail before (or after for subjects with confidential numbers whose address was unknown) the first telephone call. Eligible households were required to include at least one person in the study age group (15 to 75 years old) and speak French. Within the household, the person surveyed was randomly selected from among the eligible family members using the next-birthday method [16]. Anonymity and respect of privacy were protected by a procedure that deleted the phone numbers.

The data were weighted by the number of eligible individuals and phone lines in a household and were also adjusted for 2008 French population structure (available from the National Institute of Statistics and Economic Studies) according to age, gender, educational level, geographical region, urbanization level and phone equipment. Verbal consent from participants was asked at the survey beginning and the project obtained ethics approval from the French commission on data privacy and public liberties (CNIL - *Commission Nationale Informatique et Liberté*). As with all French telephone surveys, the participation of persons solicited was more difficult than for previous waves: refusal rate was about 40% for both samples (mobile and landline) [17]. For the questionnaire on exposure to artificial UV radiation a total of 3,359 people aged 15 to 75 years old were interviewed. The questionnaire lasted on average 36 minutes.

Statistical analyzes were performed with the software Stata (Version 10SE). The calculation of confidence intervals and comparison tests were performed using the statistic used in surveys by random selection. Chi-square test was used for comparisons of categorical variables and the variables in classes ordered with p = 0.05 for maximum significance level. A logistical regression allowed quantifying with precision relations between variables, controlling the effects of the model structure.

## Results

### Sociodemographic characteristics of people having tanning sessions during their lifetime

**Figure 1:**
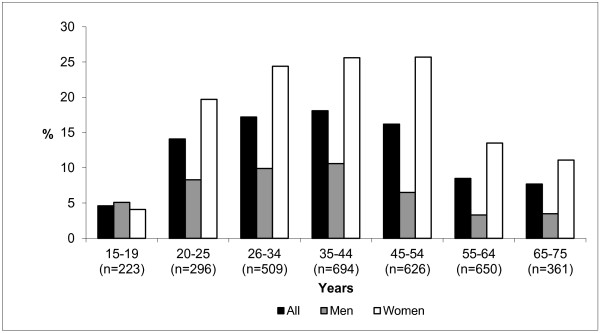
Practice of artificial UV tanning during lifetime among the 15–75 years old group by sex and age.

In total, 13.4% (n = 517) of the people surveyed reported having used artificial UV radiation during their lifetime. Such use was associated with striking sociodemographic characteristics. Gender appears to be one of the most influential factors, women have already been exposed nearly 3 times more than men (19.4% vs. 7.1%; p < 0.001). The differences in practice according to gender were observed for the age groups comprised between 20 and 75 years of age (Figure 1). After 45 years old, women are 4 times exposed than men (17.8% vs. 4.7%; p < 0,001) whereas before 45 years old they are 2 times (20.8% vs. 9.1%; p < 0,001). One alarming observation concerned use in people below the age of 18 years: although the use of UV booths is prohibited for minors, 3.5% of them reported having used one during their lifetime.

UV use is often associated with the social level of individuals. The percentage of people having used UV radiation during their lifetime increased with the income per consumption unit (29.8% for women with an income above 1,800 Euros per consumption unit). Though this was true for women (p < 0.001), the trend was not significant in men.

People without a high school degree used UV radiation less during their lifetime than others (9.9% vs. 18.3% respectively; p < 0.001).

### Exposure to artificial UV radiation over the last 12 months

In addition to data on the exposure to sunbed use at least once during a lifetime, it is important to determine current use (over the last 12 months) as well as the frequency of exposure.

**Figure 2:**
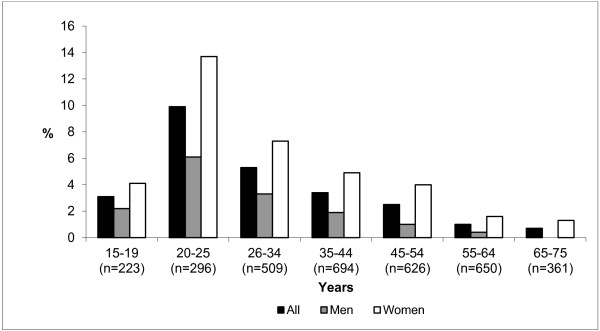
Practice of artificial tanning in the past 12 months among 15–75 years old group by sex and age.

Over the last 12 months prior to the survey, 3.5% of the people surveyed reported having used sunbed use (n = 122), which represents slightly more than a quarter of people who have been exposed to UV during their lifetime. Again here, use of this practice is more pronounced in women than in men (5.0% vs. 2.0% respectively; p < 0.001) (Figure 2). In 2010, the practice predominated in the young population of 20–25 year olds with exposure over the last 12 months of 13.7% in women compared to 6.1% in men (p < 0.05). People 25 years old and above were much less concerned. The distribution of recent exposures also followed a gradient according to income.

Among people having used sunbed over the last 12 months, we observed large variations in the frequency of use. While 19.4% were only exposed once during the year, 26.4% were exposed more than once a month. Among the 20–25 year olds, 22.2% were exposed once during the year and 26.2% were exposed more than once a month (without significant difference between the age groups).

Over the last 12 months prior to the survey, we observed the same proportion of people that used such equipment between 1 and 3 times (29.3%) and more than 10 times (32.6%). This suggests two types of behaviour that are quite distinct. The first corresponds to occasional use (fewer than 3 times a year) and the second represents regular use (more than 10 times a year).

### Places of exposure to artificial UV

One of the characteristics of sunbed use is the diversity of locations that offer this service (tanning centers, beauty salons, gyms, pools, etc.). For this reason, the (no exclusive) question regarding the places of exposure was asked to users in order to evaluate their habits (Table 1). Tanning centers and beauty salons were preferred by people having used sunbed over the last 12 months (46% and 50% of those surveyed reported having gone to these locations, respectively).Among people aged under 18 years old (n = 3), all of them used sunbed in tanning centers.

**Table 1:** Locations of exposure to artificial UV radiation (several possible responses)

***Places of exposure***	***Proportion of users (n = 122)***
Beauty salons	50%
Tanning centres	46%
Pools or spas	4.5%
Doctor’s office	3.5%
Gyms	2.6%
Home	1.5%

### Knowledge and popular misconceptions relative to cancer risks associated with UV booths

This study shows that 49.2% of the people surveyed felt that they were well informed on the risks of cancer associated with UV booths (52.7% of women vs. 45.4% of men; p < 0.001).

The people who have used sunbed over the last 12 months believe that they are better informed on the risks of cancer than people who haven’t had tanning sessions (61.7% vs. 47.7%; p < 0.05).

In total, 89.2% of the people surveyed believed that exposure to sunbed is a possible cause of cancer. The people having used sunbed during their lifetime were slightly less, in proportion, than the others to take into account the risks of cancer (85.9% vs. 89.7%; p < 0.05).

Statement: ''Using UV before going on vacations helps prepare the skin to protect it from sunburns''. 24.1% agreed with this statement, without any significant difference by gender and age. On the other hand, marked differences were observed between the group of people having used UV radiation and the group of people having never used sunbed. 42.9% of the first group agreed with this statement, compared with 21.2% in the second group (p < 0.001). Finally, there was no significant difference between sunbed regular users (more than 10 times) (48.9% agreed with this statement) compared with 61.0% among sunbed occasional users (fewer than 3 times a year).

### Determining factors of artificial UV exposure

The determining factors of UV exposure over the last 12 months were analyzed according to the level of information and popular misconceptions relative to the risks of cancer associated with artificial sunbed use, while controlling the structure effects related to sex, age and household income (Table 2). Analysis of the determining factors of exposure identified certain types of users. First of all, we found a clear difference between men and women (OR = 2.8 [1.7; 4.4], where women are much larger consumers of tanning equipment. Exposure to sunbed use is less important if age increase (OR = 0.95 [0.94; 0.96]). Furthermore, a clear social gradient was observed on the measured data. This practice seems associated with income, people with income greater than or equal to 1800 Euros by consummation unit and more are more concerned (OR = 2.3 [1.3; 4.1]). People feeling well informed (OR = 2.0 [1.3; 3.3]) and believing that sunbed use prepares the skin and avoids sunburn (OR = 4.3 [2.7; 6.7]) and do not know that UV is not a possible cause of cancer (OR = 1.7 [0.9; 3.1]) seem to favor their use.

**Table 2:** Logistic regression for the use of UV in the past 12 months (n = 3321)

**Explicative variables**	**n observed**	**% weighted**	**OR**	**CI 95%**
**Sex**		***		
man (ref.) (n = 1472)	30	2.0	- 1 -	
woman (n = 1887)	92	5.0	2,8	[1.7; 7.4]
**Age (continous)**				
(n = 3359)	122		0.95	[0.94; 0.96]
**Household Income**				
less than 1 100 Euros (ref.) (n = 921)	31	2.8	- 1 -	
1100 to 1800 Euros (n = 1156)	40	3.4	1.4	[0.8; 2.4]
1800 Euros and more (n = 1006)	41	4.5	2.3	[1.3; 4.1]
missing (n = 276)	10	3.8	1.1	[0.4; 2.9]
**Information about the risks of cancer associated with UV**		***		
poorly informed (ref.) (n = 1642)	42	2.6	- 1 -	
well informed (n = 1686)	80	4.6	2	[1.3; 3.3]
**UV exposition is a possible cause of cancer**		***		
yes (ref.) (n = 2966)	96	3.3	- 1 -	
no (n = 356)	25	5.6	1.7	[0.9; 3.1]
**Using UV before going on vacations helps prepare the skin to protect it from sunburns**		***		
don't agree (réf.) (n = 2501)	51	2.1	- 1 -	
agree (n = 820)	71	8.2	4.3	[2.7; 6.7]

*** : p < 0.001 ; ** : p < 0.01 ; * p < 0.05; results obtained by Chi^2^ test Pearson for the column % (percentage from bivariate analysis).

Hosmer-Lemeshow goodness-of-fit test:

F-adjusted test statistic = 1159.2532.

p-value = 0,000000.

## Discussion

This study presents, for the first time, data on both artificial UV radiation exposure in the French population and on the perception of the risk for cancer, as well as tanning bed use determinants. Young women, between the ages to 20 and 25 years, are a group characterized by a frequent use as 13.7% of them was exposed over the last 12 months. Nearly one out of three users exposed over the last 12 months went to UV booths more than 10 times a year.

A number of limitations of this study should be noted. First, we had a refusal rate of 40%. This rate, which is current in recent French general population surveys, could be differential regarding to some sunbed use characteristics, and in this case could influence our results. However, we expect this bias is limited because the person surveyed was randomly selected and we can assume that this refusal response was randomly distributed in the population. Second, this study characterizes only the beliefs and practices about tanning bed use, but we did not include data on physical exposure to artificial UV (UV intensity combined with exposure time). In fact, the artificial UV tanning sector has been expanding for several years in many European countries. Although it is less developed in France, this market has shown signs of significant growth over the last ten years (increase in the number of devices available and increase in the number of specialized centers) [17]. Professionals in this sector are leading a very active communication campaign to attract new and increasingly younger clients, based on non-scientifically proven information. Many popular misconceptions are spread among the population such as ''exposure to artificial UV radiation helps to prepare the skin for the sun and prevent sunburns'' or the role of artificial UV in fighting vitamin D deficiencies. Any reference to a beneficial effect for health is prohibited by law (‘Article 12 du décret n°97-617 du 30 mai 1997’). Yet, such information is commonly spread via internet and in many articles addressed to the general public. Moreover, European standard for cosmetic sunbeds, EN 60 335-2-27, states that information regarding risks of sunbed use shall be part of the users instruction [18]. These results suggest public health policies efforts in this way.

This study identified two highly distinct types of use. The first concerns people who use tanning beds occasionally. The second concerns people using such equipment frequently, often several times per month. Public health interventions will need to take into consideration the existence of both categories of users. In addition, we should highlight the existence of artificial UV use in a population below the age of 18 years, though this practice is forbidden as reported in a recent French study [19].

Regulatory control should thus be reinforced in order to put an end to the previously mentioned popular misconceptions by increasing awareness among the general population and users and informing them of the risks associated with this practice. The most important risks are health-related, such as, skin cancer, cataracts, the weakening of immune defenses and, in some cases, phototoxicity and photoallergy [4,17,20].

The aesthetic consequences of UVA exposure, such as photo-induced accelerated skin aging after 10-20 years, might also constitute a possible lever to reduce use of tanning booths. Prevention efforts could be implemented in association with recommendations for sun exposure, notably via health professionals (general physicians, dermatologists, pharmacists physiotherapists, nurses).

Moreover, other studies of this type should be conducted in the future to measure changes in prevalence, beliefs, knowledge and other tanning bed use determinants. Incorporating findings related to tanning dependency, peer group affiliation, media influences and other constructs will also improve our understanding and ability to develop efficacious interventions to reduce engagement in this health risk behavior.

A systematic review of intervention efforts to reduce indoor tanning was conducted and the authors [21] concluded that there was very limited research on indoor tanning interventions. Further studies should produce evidence based knowledge about the reduction to sunbed use exposure.

## Conclusions

This study gives an overview of the current situation regarding practices, knowledge and perception of artificial UV associated risks among the general French population and proposes some possible options for prevention of cancer risks associated with artificial UV radiation. These population exposure results will serve to study the importance of artificial UV radiation in the progression of melanoma incidence in France and in Europe. It could also be interesting to investigate the age of first use as the 2006 IARC meta-analysis showed that exposure before the age of 35 led to a higher increase in the risk of melanoma and that the risk level also increased with longer periods of exposure [4].

Therefore, exposure to artificial UV radiation is truly a public health menace and challenge put before authorities [22]. Hence, it seems relevant to provide targeted information to users concerning the risks associated sunbed use and the risks of the sun and tanning booths in particular. Furthermore, European and national regulations controlling the artificial tanning sector should be enhanced.

## Abbreviations

UV: Ultra violet; INPES: Institut National de Prévention et d’Education à la Santé; IARC: International Agency for Research on Cancer; RR: Relative risk; CATI: Computer-Assisted Telephone Interviewing; ADSL: Asymmetric digital subscriber line; INSEE: Institut National de la Statistique et des Etudes Economiques.

## Competing interest

The authors declare they have no competing financial interests.

## Authors’ contributions

All authors contributed to the development of the study aims. CL realized statistical analyses and reviewed the final manuscript. FB supervised the study and acted as guarantor. TB wrote the first and final versions of the manuscript. All authors read and approved the final manuscript.

## Pre-publication history

The pre-publication history for this paper can be accessed here:

http://www.biomedcentral.com/1471-5945/13/6/prepub
